# Timing of renal-replacement therapy in patients with acute kidney injury and sepsis: a systematic review and meta-analysis

**DOI:** 10.1186/s12882-026-05132-4

**Published:** 2026-06-18

**Authors:** Zaid Bin Sahaq, Saud Mohammed Ali Alotaibi, Maryam Alwan Mousa Ali, Ryan Khater Alanzi, Ghadeer AlHayki, Faisal Almalki, Abdullah Mohammed Alshehri, Fouad Dandal, Mohamad Al Kassih, Ahmed Ibrahim Alshanqiti, Hamad Fahad AlOtaibi, Ahmed Elaraby, Fahad S. Alrashidi

**Affiliations:** 1 College of Medicine, Alfaisal University, Riyadh, 11533 Saudi Arabia; 2 College of Medicine, Taif University, Taif, 21944 Saudi Arabia; 3 Pharmaceutical Care Department, Tathleeth General Hospital, Aseer Health Cluster (Bisha Zone), Bisha, Saudi Arabia; 4 College of Medicine, Shaqra University, Shaqra, Saudi Arabia; 5 College of Medicine, Mansoura University, Mansoura, Egypt; 6 College of Medicine, King Saud Bin Abdulaziz University for Health Sciences, Riyadh, 13216 Saudi Arabia; 7 College of Medicine, Mutah University, Alkarak, 61710 Jordan; 8 Dr. Sulaiman Al-Habib Medical Group, Emergency Department, Jeddah, Saudi Arabia; 9 College of Medicine, Taibah University, Medina, Saudi Arabia; 10 College of Medicine, University of Jeddah, Jeddah, Saudi Arabia; 11 Faculty of Medicine, Al-Azhar University, Cairo, Egypt; 12 Nephrology Department, Ad Diriyah Hospital, Third Riyadh Health Cluster, Diriyah, Saudi Arabia

**Keywords:** Sepsis, Acute kidney injury, Renal replacement therapy, Early RRT, Delayed RRT, Mortality, Meta-analysis

## Abstract

**Background:**

The optimal timing for initiating renal replacement therapy (RRT) in sepsis-associated acute kidney injury (AKI) patients remains controversial. Early RRT initiation offers benefits by preventing metabolic complications and fluid overload, while delayed initiation avoids unnecessary procedures and conserves resources. This systematic review and meta-analysis aimed to compare early versus delayed RRT strategies in adult patients with sepsis-associated AKI.

**Methods:**

We conducted a comprehensive search on PubMed, Scopus, Web of Science, and Cochrane CENTRAL from inception to February 2026. We included randomized controlled trials (RCTs) and observational cohorts comparing early versus delayed RRT initiation in patients with sepsis-associated AKI. Outcomes included 28-day and 90-day mortality, multi-organ dysfunction score (MODS), and intensive care unit (ICU) and hospital length of stay. Data were pooled using random-effects models.

**Results:**

Fifteen studies (4 RCTs, 11 retrospective cohorts) comprising 6,289 patients were included. Early RRT was associated with significant reduction in 28-day mortality compared to delayed RRT (RR 0.81, 95% CI: 0.68–0.95, *p* = 0.01; I²=31.38%). However, trim-and-fill analysis suggested potential publication bias, with adjusted estimates showing non-significance (RR 0.88, 95% CI: 0.75–1.04). No significant differences were observed for 90-day mortality (RR 0.85, 95% CI: 0.69–1.04, *p* = 0.12), MODS (MD 0.24, 95% CI: -0.94–1.42, *p* = 0.69), ICU length of stay (MD -1.76, 95% CI: -4.18–0.66, *p* = 0.15), or hospital length of stay (MD 0.34, 95% CI: -3.24–3.92, *p* = 0.85).

**Conclusion:**

Early RRT initiation in sepsis-associated AKI was associated with reduction in 28-day mortality rates but did not result in any differences of 90-days mortality rates, and was not associated with improvements in MODS, suggesting that timing alone may not substantially alter long-term outcomes in this high-risk population. These findings are driven from mixed study designs with heterogeneity among the included patients, underscoring the need for more individualized approaches to RRT initiation in the future trials.

**Trial registration:**

Not applicable.

**Supplementary Information:**

The online version contains supplementary material available at https://doi.org/10.1186/s12882-026-05132-4.

## Background

Acute Kidney Injury (AKI) is a common complication in patients hospitalized in intensive care units (ICU) for sepsis, a complication that increases the risk of mortality [1, 2]. AKI associated with sepsis is a distinct pathophysiology, and patients with AKI and septic shock varies in response to renal replacement therapy (RRT) compared to patients of AKI without septic shock [3]. Managing sepsis-associated AKI with immediate RRT is established if the patients have severe conditions such as hyperkalemia, severe metabolic acidosis, and life-threatening fluid overload. However, in the absence of such cases whether to initiate RRT, and suppose we do, the timing of the initiation is unclear [4]. Clinical studies have investigated the early RRT strategy hypothesizing the early start within 12 h after documentation of failure-stage AKI might provide superior control of uremia, early correction of acid-base, and potentially clearing the inflammatory mediators, along with preventing subsequent multi-organ failure [5].

On the other hand, 48 h delayed RRT strategy argues that starting RRT early exposes the patients to unnecessary risks and many of these patients often recover spontaneously with conservative management. Recently, two randomized controlled trials that compared early versus delayed initiation of RRT resulted in conflicting findings [6, 7]. Therefore, it remains uncertain whether starting RRT early provides any benefit, and the extent of potential risk, if any associated with delayed therapy in patients with sepsis-associated AKI is still unclear.

A Recently published meta-analysis have addressed this question coming up with no conclusive results along with significant heterogeneity between the effect estimates [8]. Therefore, in this systematic review and meta-analysis, we aimed to update the current evidence with the newly conflicting studies on the timing of the RRT in patients with AKI related sepsis.

## Methods

This systematic review and meta-analysis was conducted according to the Preferred Reporting Items for Systematic Reviews and Meta-Analysis (PRISMA) [9] and following the Cochrane Handbook of Systematic review and meta-analysis guidelines [10]. We Registered the PROSPERO Protocol with ID (CRD420261319880).

### Literature search and screening

We searched PubMed, Scopus, Web of Science (WoS), Embase and Cochrane CENTRAL from inspection to February 2026 to identify all randomized controlled trials (RCTs) and observational cohorts using the follow search term: ((CRRT OR “continuous renal replacement therapy” OR RRT) AND (AKI OR “acute kidney injury” OR “acute renal failure”) AND (sepsis OR septic OR “septic shock”) AND (timing OR early OR late OR initiation OR delay)). Detailed search strategy to each database is stated in Supplementary Table S1. We conducted backward and afterward citation analyses for the reference sections of all retrieved articles for potential inclusion of all relevant studies. We screened titles and abstracts first, followed by full-text screening to ensure a clear identification of eligible studies. Title/abstract and full-text screening were performed independently by two reviewers, and any disagreements were resolved by discussion with a third reviewer.

### Eligibility criteria and endpoints

We included all studies that included adult patients with AKI requiring RRT due to bacterial infection complication that led to sepsis, compared the early RRT strategy within 12 h of documented failure-stage AKI versus delayed 48 h RRT if no renal recovery, and reported our outcomes of interest. The outcomes were the rates of mortality at 28-days and 90-days, multi-organ dysfunction score (MODS), and length of intensive unit care (ICU) and hospital stay.

### Quality assessment

The risk of the bias assessment of RCTs was performed using Cochrane risk of bias assessment tool version 2 (ROB-2) [11]. The revised version ROB-2 tool assessed five main domains as follows: bias arising from the randomization process, bias arising from the deviation from the intended intervention, bias arising from missing outcome data, bias arising from measurement of the outcome, and bias arising from the selection of the reported result. Each domain was judged as low risk, some concerns, and high risk of bias. Additionally, we used the Newcastle-Ottawa Scale (NOS) [12] for the observational cohorts. The scale uses three main domains: Selection of study groups (0–4 stars), Comparability of groups (0–2 stars), and Ascertainment of Exposure/Outcome (0–3 stars). A score of 7–9 was considered high-quality and low risk of bias, 5–6 was considered moderate quality, and 0–4 was considered low quality. Risk-of-bias assessment done independently by two reviewers, and any disagreements were resolved by discussion with a third reviewer.

### Data extraction and statistical analysis

We used a standardized Excel sheet to extract all relevant data from the included studies. The extracted data were as follows: (1) summary characteristics of the included studies including: country, time frame, study design, sample size of septic patients, inclusion and exclusion criteria, studied outcomes, and key findings, (2) baseline characteristics of the patients included such as: sample size, males, age, patients’ comorbidities, and Kidney Disease Improving Global Outcomes (KDIGO), (3) risk of bias tools domains, and (4) measured outcomes.

Dichotomous outcomes were extracted as the frequency of events with total number of patients and were pooled as risk ratio (RR) with its 95% confidence interval (CI) using restricted maximum likelihood (REML) random-effects model. Moreover, continuous outcomes were extracted as mean, standard deviations (SDs) and total number of patients, and were pooled as standardized mean difference (SMD) with its 95% CI using the above declared model. A significant heterogeneity among the studies was determined when a *p*-value was less than 0.1 and I^2^ ≥50%. A leave-one-out sensitivity analysis was performed to rule out any significant heterogeneity between the studies. Furthermore, we used funnel plot to assess for potential publication bias. STATA 19MP software was used to perform all the statistical analyses.

## Results

### Literature search and study selection

**Fig. 1:**
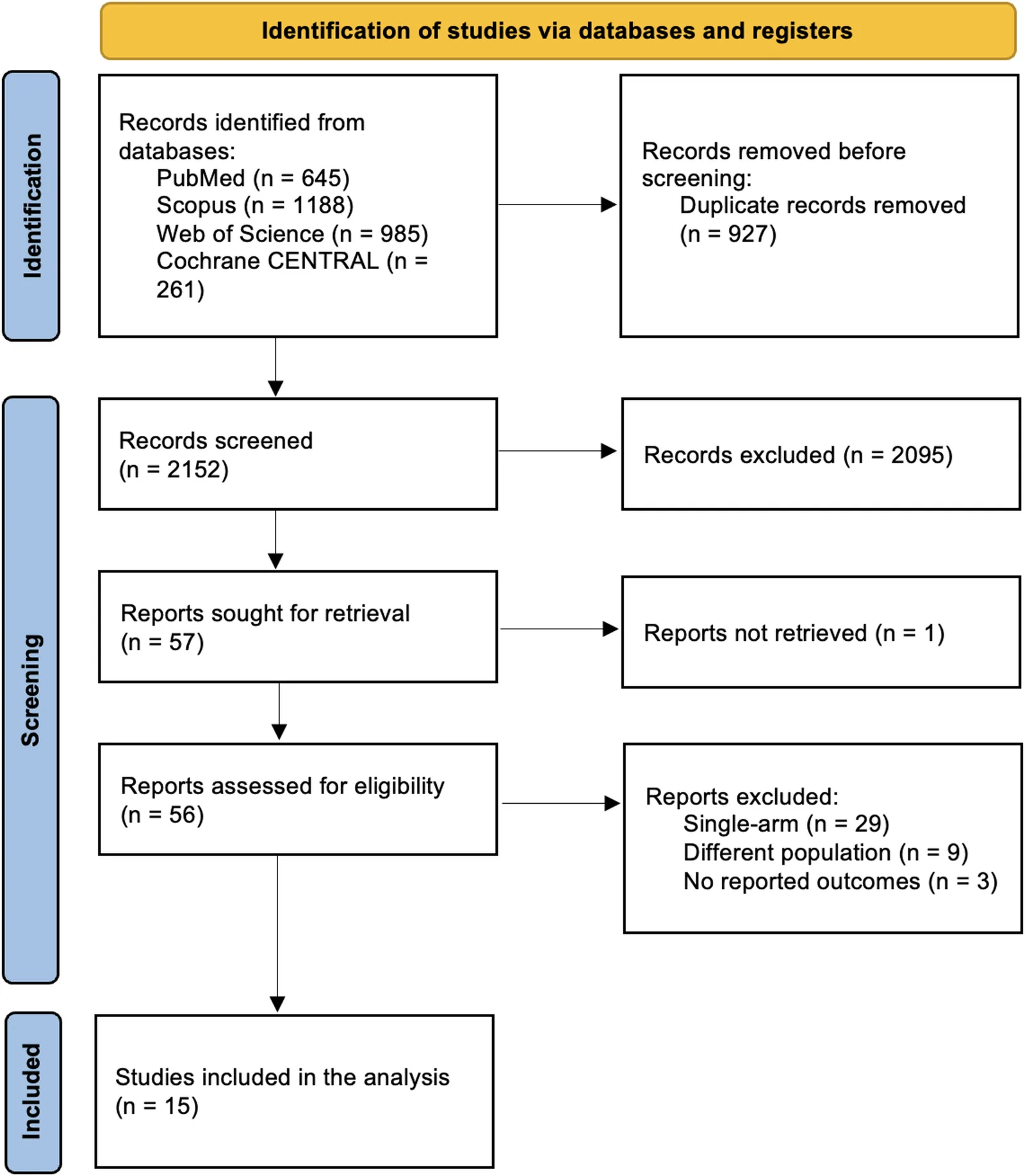
The PRISMA flow chart

Our search yielded 3,079 articles, of which 2,095 were excluded following title-abstract screening and duplicates removal leaving 56 for full-text screening. We finally included 15 studies in the meta-analysis [6, 13–26]. The selection process is shown in the PRISMA flow chart, Fig. 1.

### Study characteristics and quality assessment

The final included 15 studies were eleven observation retrospective cohorts [13, 15–18, 20–22, 24–26], three RCTs [14, 19, 23], and one post-hoc analysis of RCT [6]. The studies included a total of 6,289 patients, 32.1% (4,106 patients) were males, with mean age of 62.7 ± 14.1 years. The included studies were conducted in several countries (South Korea, China, France, India, and Hong Kong) and comprised both single-centre and multicentre studies, including the international multi-centre STARRT-AKI trial. About 3,241 (51.5%) patients received early RRT, and 3,048 (48.5%) patients received delayed RRT. More detailed summary characteristics of the included studies and baseline data of the patients are stated in Tables 1 and 2.

**Table 1:** Summary characteristics of the included studies

Study ID	Country	Design	Time frame	Septic patients	Inclusion Criteria	Exclusion Criteria	Outcomes	Key findings
Baek 2017	South Korea	Single-center retrospective cohort	Jan 2014 – Dec 2015	177	Septic shock with vasopressor use, AKI by KDIGO criteria, CRRT initiated	Chronic dialysis, Organ transplant, Stage IV malignancy, Age < 18, Referrals from other hospitals, No vasopressor use during CRRT	Primary: 28- & 90-day mortality. Secondary: ICU LOS, MV duration	CRRT within 24 h of vasopressor start associated with lower mortality
Barbar 2018	France	Multicenter RCT	Jul 2012 – Oct 2016	488	Septic shock within 48 h of vasopressor start, Severe AKI (RIFLE Failure stage), No life-threatening AKI complications	Emergency RRT needed before randomization, Age < 18, Pregnancy, Decision to limit care	Primary: 90-day mortality. Secondary: 28- & 180-day mortality, RRT-free days, ICU/Hospital LOS	No mortality difference between early (within 12 h) vs. delayed (after 48 h if no recovery) RRT, 38% in delayed group avoided RRT
Chon 2012	South Korea	Single-center retrospective cohort	Apr 2009 – Oct 2010	55	Severe sepsis/septic shock, RIFLE Injury or Failure, Age > 18, CRRT initiated	Liver cirrhosis Child-Pugh C, APACHE II > 40, SOFA > 14 at sepsis, Age > 80, Stage IV malignancy, ICU stay < 48 h, Recent cardiac arrest/shock	Primary: 28-day mortality. Secondary: 90-day mortality, MV-free days, MODS	CRRT ≤ 24 h from sepsis onset associated with lower 28-day mortality vs. >24 h, RIFLE stage not predictive of mortality
Gaudry 2018	France	Post-hoc analysis of multicenter RCT (AKIKI trial)	2013–2016	348	Severe AKI (KDIGO stage 3), Invasive MV and/or catecholamines, Subgroups: septic shock (Sepsis-3) and ARDS (Berlin definition)	Life-threatening indications for RRT, CKD stage 5	Primary: 60-day mortality. Secondary: RRT use, renal recovery, fluid balance, MV-free days	No mortality benefit with early RRT in septic shock or ARDS subgroups. Delayed strategy reduced RRT use (~ 45% avoided). Higher urine output with delayed strategy
Tian 2014	China	Single-center, Retrospective Cohort	Nov 15, 2009 – Dec 31, 2011	160	Adults (> 12 yrs) with septic AKI (2001 Intl. Sepsis Def. Conf. & AKIN criteria), Admitted to a 22-bed ICU.	Age < 12 yrs, Chronic renal illness or terminal illness, Pre-admission CRRT or CRRT for non-renal indications, CRRT duration < 24 h, ICU stay < 72 h.	Primary: 28-day mortality. Secondary: Renal recovery (dialysis-free at discharge), ICU length of stay, ventilation time.	28-day mortality increased with AKIN stage in both CRRT and control groups (*p* = 0.001 & 0.029).
Xiao 2021	China	Single-center, Retrospective Cohort	2016–2019	123	Adults (> 18 yrs) with community-acquired sepsis & AKI (KDIGO criteria), Received CRRT within 72 h of admission to medical ICU.	Chronic dialysis (*n* = 70), Previous renal transplant (*n* = 10), Cardiopulmonary arrest on admission (*n* = 2), Trauma, poisoning, transplant, pregnancy, Non-infectious diseases or secondary (nosocomial) infections.	Primary: ICU mortality. Secondary: 30-, 60-, 90-day mortality (for early vs. late groups).	Early CRRT (≤ 16 h) had significantly lower mortality at 30, 60, and 90 days vs. late group (> 16 h) (all *p* < 0.0001). Initiating CRRT within 16 h of AKI onset may reduce mortality in community-acquired septic AKI.
Yoon 2019	South Korea	Single-center, Retrospective Cohort	July 2016 – April 2018	158	Adults (≥ 18 yrs) with septic shock (Sepsis-3 criteria) & AKI (KDIGO criteria), Admitted to medical ICU and received CRRT.	Prior dialysis (*n* = 65), CKD stage 5 not on dialysis (*n* = 2), Liver failure (*n* = 11), CRRT for non-septic shock indications (*n* = 12), Sepsis without shock (*n* = 40).	Primary: ICU mortality. Secondary: 28-, 60-, 90-day mortality (for early vs. late groups).	Early CRRT (< 16.5 h) had significantly lower cumulative mortality at 28, 60, and 90 days vs. late group (≥ 16.5 h) (all *p* < 0.001). Initiating CRRT within 16.5 h of AKI onset may improve survival in septic shock patients.
Oh 2012	South Korea	Retrospective cohort	Sep 2009 – Feb 2011	210	Septic AKI, CRRT, ↑ vasopressor dose in first 6 h	Age < 18, chronic dialysis, advanced liver disease, non-septic AKI, multiple AKI episodes	28-day mortality	Early CRRT (≤ 2 days after vasopressor start) decreases mortality (HR 0.548, *p* = 0.02)
Oh 2016	South Korea	Retrospective observational	Jun 2008 – Feb 2013	60	Septic AKI, ED admission, EGDT protocol, CRRT	Age < 18, cirrhosis Child-Pugh C, chronic dialysis, APACHE II > 40, stage IV cancer	28-day mortality	Early CRRT (≤ 26.4 h after EGDT) decreases mortality (HR 2.461, *p* = 0.026)
Shum 2013	Hong Kong, China	Retrospective cohort	Jan 2008 – Jun 2011	120	Septic shock + AKI (sRIFLE Risk/Injury/Failure), CRRT	Age < 18, CKD stage 5, long-term dialysis, pre-ICU RRT	SOFA score change, mortality (28d, 3 m, 6 m), dialysis dependence	No benefit of early CRRT (by sRIFLE) in SOFA improvement or mortality
Bagshaw 2020	Multinational	RCT	2019–2020	1689	Critically ill with severe AKI, clinical equipoise for RRT timing	Emergency RRT needed, imminent kidney recovery expected, > 12 h from eligibility	90-day mortality, RRT dependence, adverse events	No mortality difference; increased RRT dependence and adverse events with early RRT
Julien 2025	France	Target Trial Emulation (Observational)	2010–2020	295	Documented leptospirosis, KDIGO Stage 3 AKI on admission • No immediate RRT need	Stage 1–2 AKI, Immediate RRT indication	Primary: Composite of death or new/worsening CKD at 1 year. Secondary: Mortality, CKD incidence	Early RRT (≤ 48 h) associated with higher risk of death/CKD (OR 2.08, *p* = 0.046). No mortality benefit.
Lee 2024	South Korea	Retrospective Cohort	2008–2019	225	Sepsis-3 criteria, KDIGO Stage 2–3 AKI, CKRT within 48 h of AKI	Death within 48 h, Prior dialysis history	Primary: 28-day mortality. Secondary: 90-day mortality, ICU/hospital stay, organ support-free days	Early CKRT (≤ 6 h) reduced 28-day mortality (26.7% vs. 43.9%, *p* = 0.035) and increased ventilator/vasopressor-free days.
Maiwall 2025	India	RCT	Jan 2018 - Sep 2019	50	Cirrhosis with septic shock, KDIGO Stage 2–3 AKI after fluid resuscitation	Age < 18, CKD, HRS-AKI, Absolute dialysis criteria at baseline, Refractory shock	Primary: 28-day mortality. Secondary: Shock reversal, renal recovery, IDH, biomarkers	Early SLED (≤ 12 h) reduced early deaths (Day 7: 20% vs. 52%, *p* = 0.038), increased renal recovery (68% vs. 12%, *p* < 0.001) and shock reversal (60% vs. 16%, *p* = 0.001).
Li 2026	China	Retrospective Cohort	2008–2022	2,131	Sepsis-3 criteria, KDIGO AKI, Oliguria (< 0.5 mL/kg/h ×6 h), CRRT records	Multiple ICU admissions, Missing CRRT data, ICU stay < 24 h	Primary: 90-day mortality. Secondary: Hospital mortality, ICU stay	Early CRRT (≤ 48 h after oliguria) improved 90-day survival (ATE 0.11, *p* = 0.01) and reduced hospital mortality (HR 2.06, *p* < 0.001).

AKI; Acute kidney injury, CRRT; Continuous renal replacement therapy, RRT; Renal replacement therapy, RCT; Randomized controlled trial, LOS; Length of stay, MV; Mechanical ventilation, MODS; Multiple organ dysfunction score, KDIGO; Kidney Disease Improving Global Outcomes, RIFLE; Risk, Injury, Failure, Loss, End-stage kidney disease, SOFA; Sequential Organ Failure Assessment (organ dysfunction score), CKD; Chronic Kidney Disease, IDH; Intradialytic Hypotension, mITT; modified intention-to-treat, EGDT; Early Goal-Directed Therapy, SLED; Sustained Low-Efficiency Dialysis, ATE; Average Treatment Effect, HR; Hazard Ratio

**Table 2:** Baseline characteristics of the included patients

Study	Group	Patients	Male	Age, years	DM	CHD	CKD	CLF	Lactate	KDIGO		HR	AKI		
										Stage 3	Stage 2		I	II	III
Baek 2017	Early	125	71 (56.8)	67.8 ± 14.4	NR	NR	NR	NR	NR	NR	NR	NR	23 (18.4)	46 (36.8)	56 (44.8)
	Late	52	34 (65.4)	66.6 ± 12.8	NR	NR	NR	NR	NR	NR	NR	NR	13 (25)	17 (32.7)	22 (42.3)
Barbar 2018	Early	246	142 (58)	69.3 ± 11.6	80 (33)	9 (4)	32 (13)	31 (13)	NR	NR	NR	NR	0 (0)	0 (0)	246 (100)
	Late	242	154 (64)	68.7 ± 12.8	69 (29)	9 (4)	44 (18)	31 (13)	NR	NR	NR	NR	0 (0)	0 (0)	242 (100)
Chon 2012	Early	36	24 (66.7)	63 ± 9.9	NR	NR	NR	NR	4.5 ± 3.1	NR	NR	NR	19 (52.8)	17 (47.2)	NR
	Late	19	14 (73.7)	61.7 ± 12	NR	NR	NR	NR	3.1 ± 2.6	NR	NR	NR	11 (57.9)	8 (42.1)	NR
Gaudry 2018	Early	174	113 (65)	67 ± 13	46 (26)	NR	NR	NR	NR	NR	NR	NR	NR	NR	NR
	Late	174	106 (61)	68 ± 12	46 (26)	NR	NR	NR	NR	NR	NR	NR	NR	NR	NR
Tian 2014	Early	100	79 (79)	51.7 ± 18.9	NR	NR	0 (0)	NR	NR	46 (46)	31 (31)	NR	23 (23)	31 (31)	46 (46)
	Late	60	44 (73.3)	57.8 ± 17.9	NR	NR	0 (0)	NR	NR	13 (21.7)	21 (35)	NR	26 (43)	21 (35)	13 (22)
Xiao 2021	Early	72	72 (58.5)	60 ± 25.5	NR	8 (4.9)	35 (28.5)	NR	5.1 ± 9.5	NR	NR	111 ± 25.2	NR	NR	NR
	Late	51													
Yoon 2019	Early	93	61 (65.6)	70.5 ± 20.8	NR	NR	35 (37.6)	NR	9.1 ± 9.5	93 (100)	0 (0)	115.5 ± 11.3	0 (0)	0 (0)	93 (100)
	Late	65	36 (55.4)	69.0 ± 20.8	NR	NR	18 (27.7)	NR	9.1 ± 9.5	65 (100)	0 (0)	115.5 ± 11.3	0 (0)	0 (0)	65 (100)
Oh 2012	Early	105	69 (65.7)	62.3 ± 12.9	47 (44.8)	18 (17.1)	NR	NR	NR	30 (28.6)	36 (34.3)	NR	39 (37.1)	36 (34.3)	30 (28.6)
	Late	105	57 (54.3)	62.5 ± 15.4	14 (13.3)	16 (15.2)	NR	NR	NR	29 (27.6)	44 (41.9)	NR	32 (30.5)	44 (41.9)	29 (27.6)
Oh 2016	Early	30	26 (86.7)	63.6 ± 14.6	14 (46.7)	9 (30)	NR	3 (10)	6.3 ± 4.4	24 (80)	5 (16.7)	NR	1 (3.3)	5 (16.7)	24 (80)
	Late	30	26 (86.7)	69.1 ± 13.4	7 (23.3)	8 (26.7)	NR	5 (16.7)	5.4 ± 4.4	18 (60)	8 (26.7)	NR	4 (13.3)	8 (26.7)	18 (60)
Shum 2013	Early	31	21 (67.7)	74 ± 11.85	NR	NR	NR	NR	NR	NR	NR	NR	NR	NR	NR
	Late	89	56 (62.9)	73 ± 14.81	NR	NR	NR	NR	NR	NR	NR	NR	NR	NR	NR
Bagshaw 2020	Early	1465/855*	995 (67.9)	64.6 ± 14.3	439 (30)	320 (21.8)	658 (44.9)	172 (11.7)	NR	NR	NR	NR	NR	NR	NR
	Late	1462/834*	995 (68)	64.7 ± 13.4	459 (31.4)	328 (22.4)	626 (42.8)	165 (11.3)	NR	NR	NR	NR	NR	NR	NR
Julien 2025	Early	82	77 (94)	52 ± 16.3	NR	NR	NR	NR	NR	NR	NR	106 ± 18.9	NR	NR	NR
	Late	213	200 (94)	51 ± 12.6	NR	NR	NR	NR	NR	NR	NR	96 ± 18.5	NR	NR	NR
Lee 2024	Early	45	30 (66.7)	62 ± 17.8	18 (40)	NR	13 (28.9)	17 (37.8)	5.3 ± 5.1	36 (80)	9 (20)	NR	NR	NR	NR
	Late	180	115 (63.9)	61 ± 13.3	73 (40.6)	NR	49 (27.2)	74 (41.1)	6.1 ± 4.7	141 (78.3)	39 (21.7)	NR	NR	NR	NR
Maiwall 2025	Early	25	23 (92)	45.2 ± 10	NR	NR	NR	NR	2.7 ± 1.8	25 (100)	0 (0)	118 ± 21	0 (0)	0 (0)	25 (100)
	Late	25	22 (88)	45.2 ± 10	NR	NR	NR	NR	3.3 ± 2.1	25 (100)	0 (0)	119 ± 20	0 (0)	0 (0)	25 (100)
Li 2026	Early	1222	736 (60.2)	62.1 ± 14.9	NR	NR	NR	NR	6.01 ± 5.21	1172 (95.9)	3 (0.25)	112 ± 23.9	47 (3.85)	3 (0.25)	1172 (95.9)
	Late	909	551 (60.6)	65.3 ± 14.5	NR	NR	NR	NR	3.67 ± 3.19	874 (96.1)	8 (0.88)	109 ± 23.2	27 (2.97)	8 (0.88)	874 (96.1)

data present as; n (%), mean ± SD, DM; diabetes mellitus, CHD; chronic heart disease, CKD; chronic kidney disease, CLF; chronic liver failure, HR; heart rate, KDIGO; Kidney Disease Improving Global Outcomes, AKI; acute kidney injury

*855 of 1465 and 834 of 1462 patients were presented with septic-associated AKI and were included in the analysis, the baseline data is for the total population

After assessing the four included RCTs using ROB-2 tool, three studies had some concern of bias due to deviation from intended intervention, and measurement of the outcome, while only one had low risk of bias (Supplementary Figure S1). The observational studies were assessed using NOS scale and all the included cohort had high-quality scores (Supplementary Table S2).

### Primary outcome

**Fig. 2:**
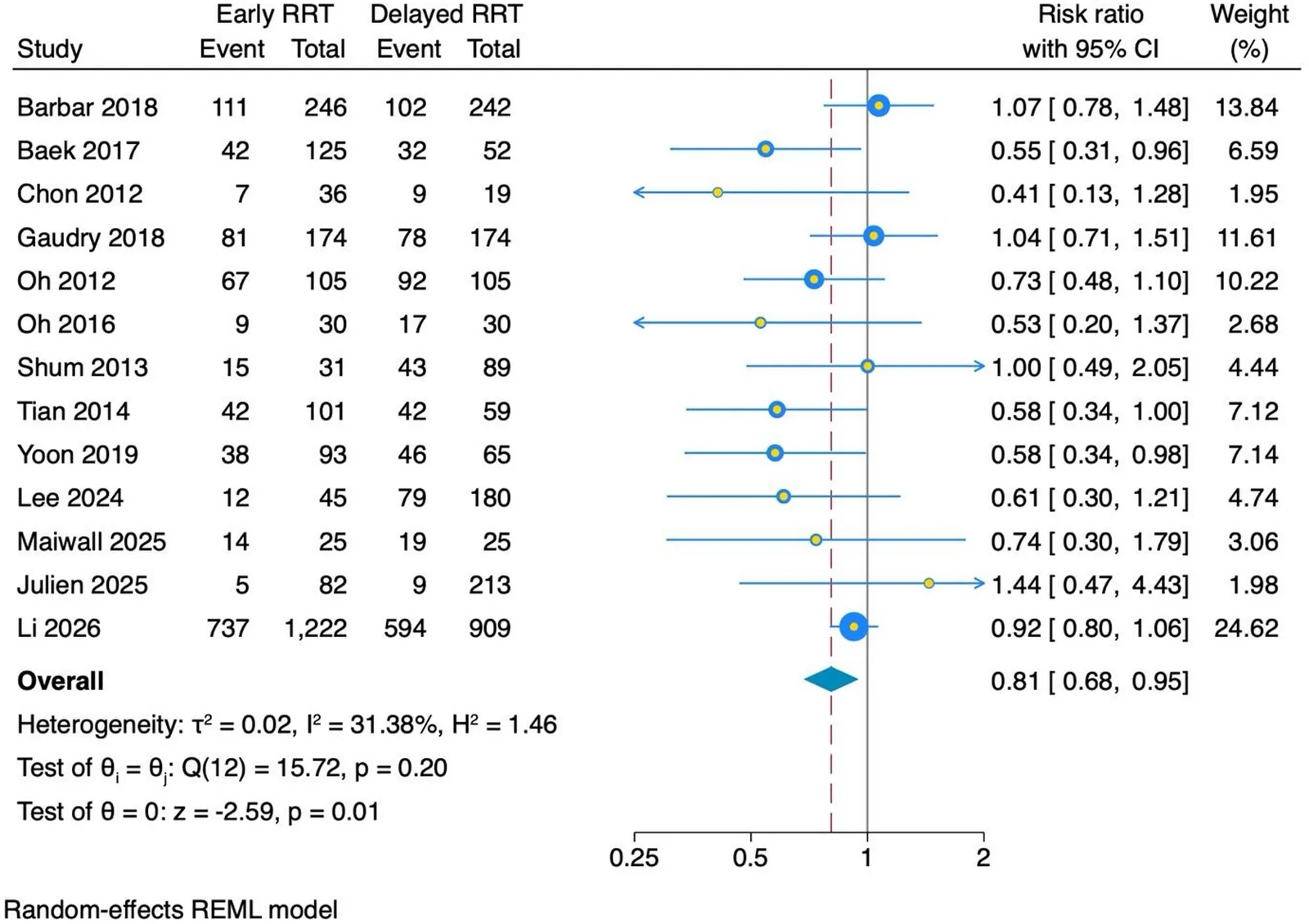
Random-effect meta-analysis of 28-days mortality rates

The incidence of ***28-days mortality*** was reported by 13 studies, in which the rates were comparable between the patients who received early RRT and those who received delayed RRT, 50.97% (1,180 of 2,315 patients) vs. 53.7% (1,162 of 2,162 patients), respectively. However, the pooled analysis showed a significant reduction by 19% in 28-days mortality rate between the patients received early RRT (RR 0.81, 95% CI: 0.68–0.95, *p* = 0.01; I^2^ = 31.38%, *p* = 0.20), as shown in Fig. 2. To test any potential publication bias, we conducted funnel plot and no studies were visualized outside the 95% precision area, however, by inspection there was asymmetry between number of the studies in the left side of the effect size (Supplementary Figure S2). We then performed trim-and-fill analysis, and it identified four imputed studies on the right side of the funnel plot to achieve symmetry (Supplementary Figure S3). The adjusted pooled RR was 0.88 (95% CI: 0.75–1.04) compared to RR 0.81 (95% CI: 0.68–0.95), which suggest potential publication bias of the included studies. Moreover, we performed leave-one-out sensitivity analysis, and no single study had disproportional effect over the pooled RR (Supplementary Figure S4).

**Fig. 3:**
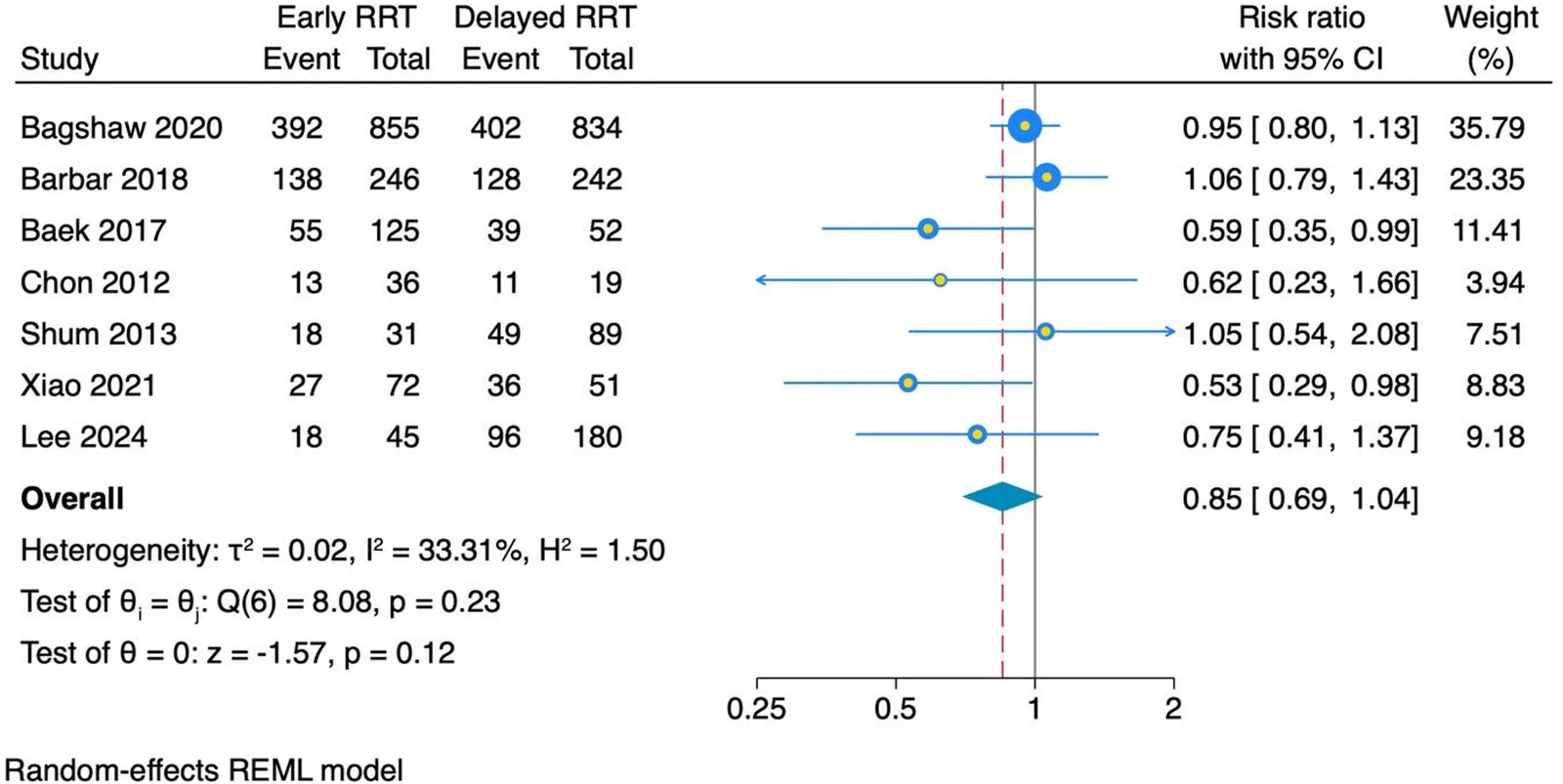
Random-effect meta-analysis of 90-days mortality rates

Seven studies reported the rates of ***90-days mortality***, of which the rate was 46.9% (661 of 1,410 patients) in the early RRT group compared to 51.9% (761 of 1,467 patients) in the delayed RRT group. The pooled estimate showed borderline no significant difference between the two RRT strategies to reduce 90-days mortality rates (RR 0.85, 95% CI: 0.69–1.04, *p* = 0.12; I^2^= 33.31%, *p* = 0.23), as shown in Fig. 3.

**Fig. 4:**
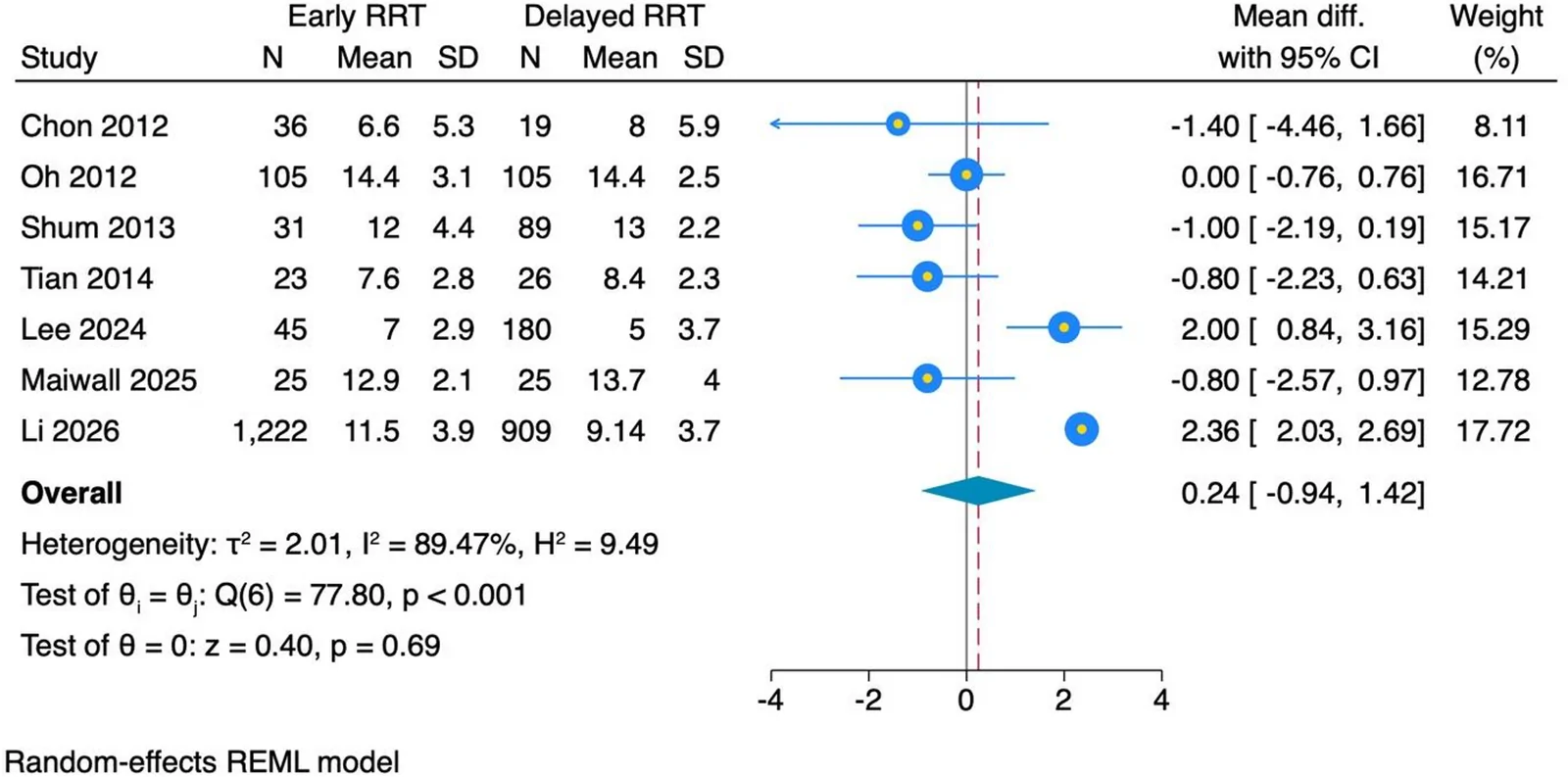
Random-effect meta-analysis of multi-organ dysfunction score (MODS)

***MODS*** were reported by seven studies, of which the pooled estimate showed no significant results between early and delayed RRT to reduce MODS (MD 0.24, 95% CI: -0.94–1.42, *p* = 0.69; I^2^= 89.47%, *p* < 0.001), as shown in Fig. 4. The leave-one-out sensitivity analysis showed no single study had disproportional effect over the pooled estimate (Supplementary Figure S5).

**Fig. 5:**
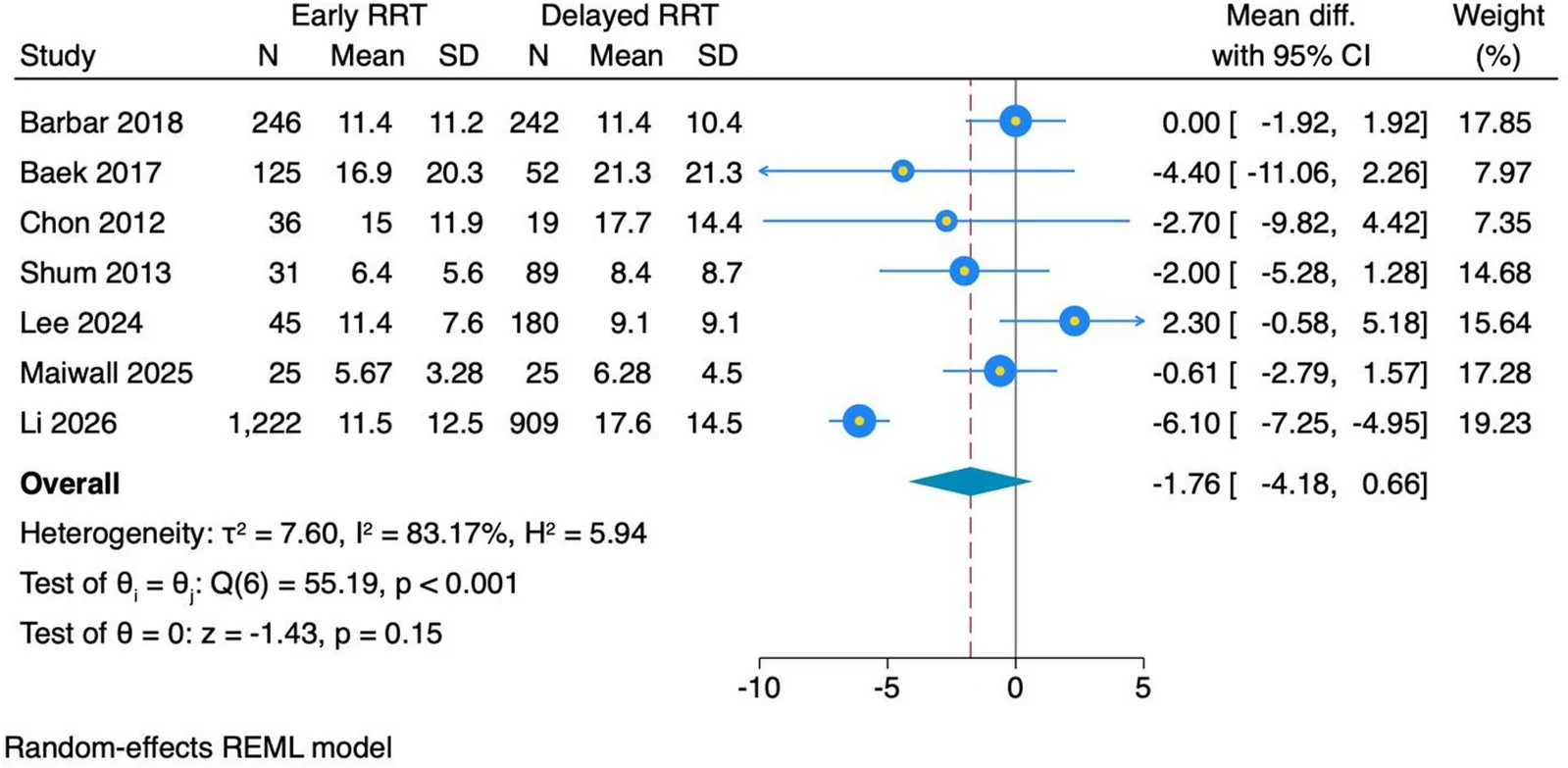
Random-effect meta-analysis of length of ICU stay

***Length of ICU*** stay was reported by seven studies, of which the pooled estimate showed no significant difference between the two studies group (MD -1.76, 95% CI: -4.18–0.66, *p* = 0.15; I^2^= 83.17, *p* < 0.001), as shown in Fig. 5. However, the leave-one-sensitivity analysis showed that by excluding *Lee et al.* there is a significant reduction in ICU stay among patients in the early RRT by 2.51 days compared to those in delayed RRT group (MD -2.51, 95% CI: -4.92 – -0.11, *p* = 0.04) (Supplementary Figure S6).

**Fig. 6:**
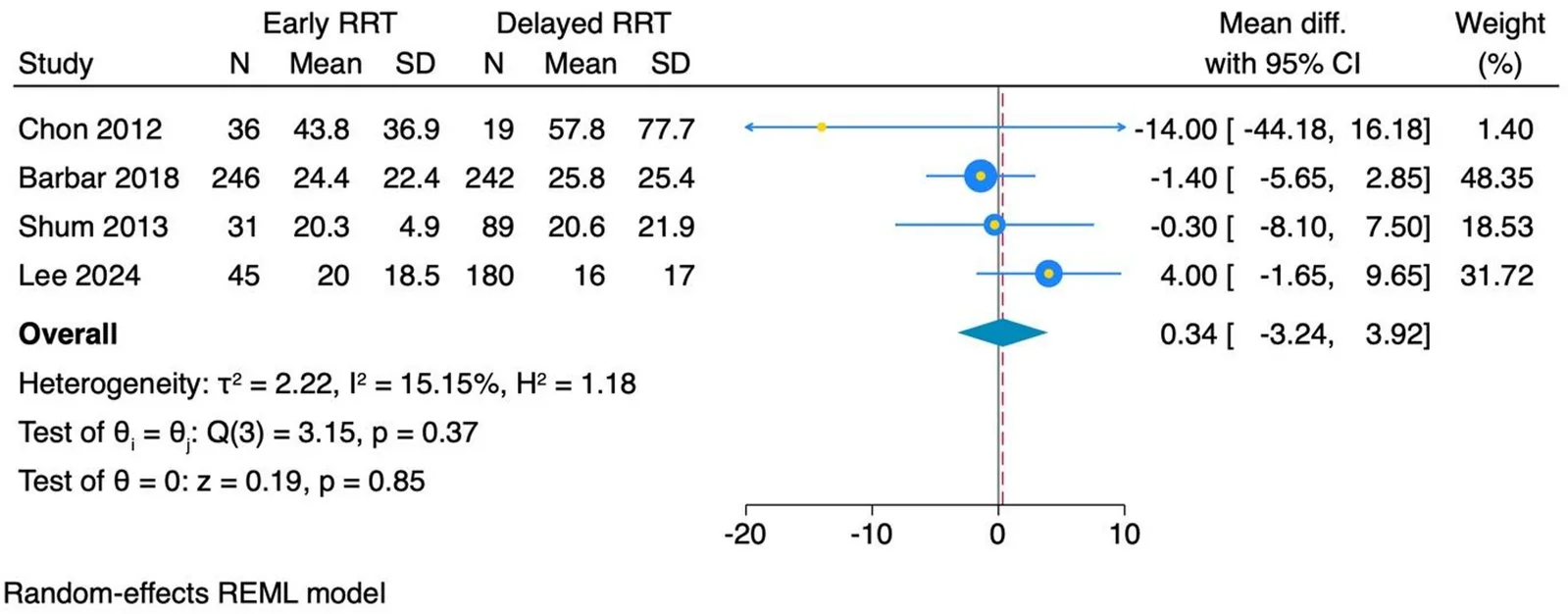
Random-effect meta-analysis of length of hospital stay

***Length of hospital stay*** was reported by only four studies, of which the pooled analysis showed no significant difference in length of hospital stay between the patients in early or delayed RRT (MD 0.34, 95% CI: -3.24–3.92, *p* = 0.85; I^2^= 15.15, *p* = 0.37), as shown in Fig. 6.

## Discussion

Our updated meta-analysis is addressing the timing of RRT in patients with AKI and sepsis. This review of 6,289 patients undergoing either early RRT or delayed RRT concluded that the incidence of 28-days mortality was significantly lower by 19% in the early RRT groups compared with delayed RRT groups. However, the pooled analysis showed none of the 90-days mortality, MODS, Length of ICU stay, or Length of hospital stay outcomes to be significantly different between the two intervention groups. The combination of severe sepsis and AKI carries a particularly bad prognosis; the mortality rate for these patients can reach as high as 70%, which is markedly higher than the 40% to 45% mortality generally observed in patients with AKI alone [2, 3]. Because of these fundamental pathophysiological differences, patients with sepsis-associated AKI often have a different physiological response to interventions such as RRT. Delaying RRT provides a window for the kidneys to spontaneously recover through conservative medical management and fluid strategies. Furthermore, delaying RRT protects patients from the harmful complications associated with this invasive therapy. Initiating RRT too early unnecessarily exposes patients to life-threatening adverse events, including catheter-related bloodstream infections, bleeding, thrombosis, and intradialytic hypotension. In septic patients who are already hemodynamically unstable, performing ultrafiltration via RRT can exacerbate renal ischemia and inflict further damage with a process sometimes referred to as “Artificial Kidney–Induced Kidney Injury”. Moreover, aggressively starting RRT early may actually compromise the body’s natural kidney repair processes, as patients subjected to early RRT strategies have demonstrated a higher risk of remaining permanently dependent on dialysis long-term compared to those managed with a delayed approach [27].

Although clinical standards often favor delaying Renal Replacement Therapy (RRT) to minimize risks, researchers initially proposed an “early” initiation strategy based on several compelling physiological hypotheses. A primary rationale was that early RRT could function as a proactive blood purification tool during the initial pro-inflammatory phase of sepsis. By filtering out the massive cascade of inflammatory mediators (the “cytokine storm”) and uremic toxins, clinicians hoped to dampen the dysregulated immune response, stop the progression of multiple-organ failure, and prevent severe complications like metabolic encephalopathy and gastric hemorrhage before the kidneys completely failed. Rather than waiting for life-threatening emergencies to start treatment, early RRT could swiftly restore acid-base homeostasis, correct electrolyte imbalances, and prevent deleterious fluid accumulation. Because sepsis and septic shock cause severe cardiovascular compromise, this management of fluid volume and inflammatory mediators was expected to promote faster hemodynamic stabilization. This strategy aimed to extend the “golden hour” concept of rapid sepsis management directly to kidney support, intervening aggressively to prevent further ischemic damage, protect vital organs, and improve overall survival.

In patients with sepsis-associated AKI the timing of RRT presents a complex clinical dilemma. With the publications that were conflicting in results, our pooled analysis shows that early RRT within 12 h of sepsis-associated AKI documentation was associated with reduction in 28-days mortality compared to delayed 48 h RRT if no renal recovery. Most of included studies consistently reported the same result with the early initiation of RRT significantly reducing the mortality at 28-days, except for *Shum et al.*, where they compared 31 patients undergoing early RRT to 89 undergoing delayed RRT and came up with no significant differences between the two arms, This likely occurred because patients in the delayed group had a higher disease severity and deteriorated much more rapidly, prompting faster intervention where they ended up being sooner to receive the RRT (median 10.8 h) than the early group (median 20.7 h) [22]. In contrast to the pooled synthesis of *Barber et al.* IDEAL-ICU trial [14] conducted in France showed no significant differences between the two groups in terms of 28-days mortality, however, they reported 239 (97%) patients in the early group had RRT and only 149 (62%) patients in the delayed group indicating that 30% of patients avoided unnecessary RRT and recovered spontaneously. This was also reported by large multicenter START-AKI trial [27] where they tested early RRT which was carried out for KDIGO stage 2 AKI patients versus delayed RRT that was started if life-threatening complication or persistent AKI for at least 72 h, they concluded that there was no significant difference in mortality between the 2 strategies yet they reported similar proportion of patients recovering spontaneously in the delayed group. Also, a greater number of patients were dependent on RRT at 90-days mark in the early RRT [27].

In Gaudry et al., [6] conducted a post-hoc analysis on the AKIKI Randomized controlled trial [28] where they analyzed 348 patients with sepsis-associated AKI randomized to either early or delayed RRT, their data showed no difference in terms of survival benefit between the two groups, however they also mentioned that 55% of patients on the delayed group underwent RRT compared to 99% of patients on the early group, pointing that the delayed RRT strategy significantly reduced the use of RRT. The data also showed that delayed RRT was associated with more cumulative urine output during the 2 days following randomization along with rapid recovery of renal function [6]. That’s why, while we got a statistically significant reduction in mortality for the short term, the large, controlled trials reported that both timings are similar in terms of mortality.

The optimal timing of RRT in patients with AKI in the context of sepsis has been debated for many years. Early RRT was hypothesized to reduce the systemic inflammation via removal of the circulating inflammatory cytokines and toxins during early peak of the sepsis so humbling the immune system response. However, with the recent randomized controlled trials [6, 14, 23] this hypothesis was questioned. A subgroup analysis based on the study type revealed a controversial result, based on the data from RCTs starting RRT early, at KDIGO stages 2 or 3, has no added benefit to the patient in terms of survival. In contrast it might increase the risk of dependence on RRT and may compromise renal recovery as shown in the early RRT arms in both IDEAL-ICU and the AKIKI post-hoc [6]. On the other hand, most observational and retrospective cohorts reported significant survival benefit associated with early RRT initiation [21, 25, 26]. This discrepancy is likely rooted in core limitations of the methodology of observational studies as we expected the covariates and hidden actors may reduce the accuracy of the results of such studies, and selection biases accompanied by retrospective designs.

The operational definition of “early” RRT varied across the included studies ranging from initiation within hours of vasopressor start or at KDIGO stage 2, to fixed time windows after AKI documentation and differed between the randomized trials and the observational cohorts. This heterogeneity in definitions limits direct comparability across studies and may partly explain the divergent results between study designs.

This finding is consistent with the recent individual-patient-data meta-analysis of RCTs [29], which aggregated data on 1,879 patients randomly allocated to early RRT or delayed RRT where patients were severe AKI, their pooled analysis showed no significant difference between the early RRT group (355 [43%] of 827) and the delayed RRT group (366 [44%] of 837) in terms of mortality at 28-days and beyond. In the absence of urgent indication for RRT, delaying the initiation of RRT, with strict patient monitoring may lead to spontaneous renal recovery and minimize the risk of RRT complications along with saving health resources [29]. The question of optimal KRT timing is not unique to sepsis-associated AKI. Across general AKI populations, randomized and observational evidence has likewise failed to demonstrate a survival advantage for accelerated initiation: no mortality difference was found among three different KRT-initiation strategies [30], a Bayesian reanalysis of the STARRT-AKI trial showed a low probability of clinically important benefit from accelerated initiation [31], and a systematic review and meta-analysis of accelerated versus waiting strategies reached the same conclusion [32]. The consistency of these findings across AKI etiologies suggests that the absence of benefit from early initiation we observed is not specific to sepsis. Sepsis-associated AKI is pathophysiologically distinct, and whether a subgroup defined by septic-AKI severity benefits from earlier initiation remains unresolved and need dedicated trials.

Beyond conventional renal indications, KRT in sepsis has also been proposed as a platform for extracorporeal blood purification that aiming to remove endotoxin and circulating cytokines, a rationale that distinguishes sepsis-associated AKI from other AKI etiologies. The 2021 Surviving Sepsis Campaign guidelines, however, do not recommend the routine use of blood purification techniques outside of clinical trials [33]. Consistent with this, a recent network meta-analysis of 60 randomized trials found that, although certain modalities such as polymyxin-B hemoperfusion and plasma exchange showed possible mortality signals, the certainty of evidence was low to very low and no modality reduced AKI incidence or KRT requirement [34]. Thus, while the immunomodulatory rationale for early KRT in sepsis is biologically appealing, it is not yet supported by high-quality evidence, reinforcing our central finding that the timing of initiation alone does not improve outcomes.

## Clinical implications

Although our pooled results showed a statistically appearant reduction in 28-day mortality with early RRT, this finding should be interpreted with caution and does not, on its own, justify routine early initiation. The signal was confined to the short-term outcome: it was not accompanied by any benefit in 90-day mortality, MODS, or length of stay; it was driven largely by the observational studies; and it lost statistical significance effect after trim-and-fill adjustment for publication bias. When restricted to the randomized trials, early RRT conferred no survival benefit and was associated with greater RRT dependence and exposure of patients to unnecessary procedures. Taken together, the totality of the evidence supports a delayed strategy with close clinical monitoring in the absence of urgent indications, since a substantial proportion of patients recover renal function spontaneously and are thereby spared the risks of RRT.

## Limitations

Although this meta-analysis provides the most updated and comprehensive synthesis of the current literature, several limitations must be acknowledged to contextualize the study findings. First limitation is reflecting the limitation of the primary literature, the lack of enough high-quality larger samples of RCTs addressing this question which warranted the inclusion of observational studies. Although the observational studies were assessed as good quality using the Newcastle–Ottawa Scale which is a validated and widely used tool for cohort studies in meta-analyses observational designs generally introduce unavoidable confounders, which we accounted for by analyzing study design as a subgroup. Furthermore, the lack of standardized procedure, since variations in the treatment modality were noticed within the studies using different techniques such as continuous veno-venous hemodiafiltration (CVVHDF), continuous veno-venous hemodialysis (CVVHD), and sustained low-efficiency dialysis (SLED) as well as during the procedure parameters including blood flow rates, dialysate flow rates, and target effluent doses.

## Conclusion

Management of sepsis-associated AKI in the ICU remains a challenge. In our pooled results, early RRT was associated with lower 28-day mortality, but this short-term signal was notapproved: it disappeared after adjustment for publication bias, was driven mainly by observational studies, and was not reproduced in 90-day mortality or in the randomized-trial subgroup, where early initiation conferred no survival benefit. Conversely, a delayed strategy spared a third of patients from RRT through spontaneous renal recovery and was associated with fewer RRT-dependent patients. So we conclude that, in the absence of urgent indications, a delayed RRT strategy with close clinical monitoring is reasonable, and that the timing of initiation alone is unlikely to substantially change outcomes in this high-risk population.

## Supplementary Information

Below is the link to the electronic supplementary material.


Supplementary Material 1


## Data Availability

No datasets were generated or analysed during the current study.

## Abbreviations

AKI: Acute Kidney Injury
CRRT: Continuous Renal Replacement Therapy
CVVHD: Continuous Veno-Venous Hemodialysis
CVVHDF: Continuous Veno-Venous Hemodiafiltration
ICU: Intensive Care Unit
KDIGO: Kidney Disease Improving Global Outcomes
MODS: Multi-Organ Dysfunction Score
NOS: Newcastle-Ottawa Scale
RRT: Renal Replacement Therapy
SLED: Sustained Low-Efficiency Dialysis
