# Supplementary material for: Timing of renal-replacement therapy in patients with acute kidney injury and sepsis: a systematic review and meta-analysis
Source: BMC Nephrol. 2026 Jun 18;27:514. doi: 10.1186/s12882-026-05132-4 (PMC13528064; doi:10.1186/s12882-026-05132-4)
Supplement: Supplementary file 1 [file 12882_2026_5132_MOESM1_ESM.docx]

**Supplementary Appendix**

| ***Content*** | ***Page*** |
| --- | --- |
| **Supplementary Table S1.** Detailed search strategy for each database. | **2** |
| **Supplementary Table S2.** The NOS assessment scale for the included cohort studies. | **3** |
| **Supplementary Figure S1.** The ROB-2 tool assessment for the included RCTs. | **4** |
| **Supplementary Figure S2.** Funnel plot analysis of 28-days mortality. | **5** |
| **Supplementary Figure S3.** Trim-and-fill analysis plot of 28-days mortality. | **6** |
| **Supplementary Figure S4.** Leave-one-out sensitivity analysis of 28-days mortality. | **7** |
| **Supplementary Figure S5.** Leave-one-out sensitivity analysis of multi-organ dysfunction socre (MODS). | **8** |
| **Supplementary Figure S6.** Leave-one-out sensitivity analysis of intensive care unit (ICU) stay. | **9** |

**Supplementary Table S1.** Detailed search strategy for each database.

| **Database** | **Search keywords** | **Filters** | **Results** |
| --- | --- | --- | --- |
| **PubMed** | (("renal replacement therapy"[Mesh] OR "continuous renal replacement therapy"[tiab] OR CRRT[tiab] OR hemodialysis[tiab] OR haemodialysis[tiab] OR hemofiltration[tiab] OR haemofiltration[tiab] OR RRT[tiab]) AND ("acute kidney injury"[Mesh] OR AKI[tiab] OR "acute renal failure"[tiab] OR ARF[tiab] OR "acute kidney failure"[tiab]) AND ("sepsis"[Mesh] OR septic[tiab] OR sepsis[tiab] OR "septic shock"[tiab]) AND ("time factors"[Mesh] OR timing[tiab] OR early[tiab] OR late[tiab] OR initiation[tiab] OR "initiation time"[tiab] OR "start time"[tiab] OR delay[tiab])) | English language | N= 645 |
| **Scopus** | TITLE-ABS-KEY ( ( "renal replacement therapy" OR "continuous renal replacement therapy" OR crrt OR rrt OR hemodialysis OR haemodialysis OR hemofiltration OR haemofiltration ) AND ( "acute kidney injury" OR aki OR "acute renal failure" OR arf ) AND ( sepsis OR septic OR "septic shock" ) AND ( timing OR early OR late OR initiation OR "initiation time" OR "start time" OR delay ) ) | English language | N= 1188 |
| **Web of Science** | TS=((("renal replacement therapy" OR "continuous renal replacement therapy" OR CRRT OR RRT OR hemodialysis OR haemodialysis OR hemofiltration OR haemofiltration) AND ("acute kidney injury" OR AKI OR "acute renal failure" OR ARF) AND (sepsis OR septic OR "septic shock") AND (timing OR early OR late OR initiation OR "initiation time" OR "start time" OR delay))) | English language | N= 985 |
| **Cochrane CENTRAL** | ("renal replacement therapy" OR "continuous renal replacement therapy" OR CRRT OR RRT OR hemodialysis OR haemodialysis OR hemofiltration OR haemofiltration) AND ("acute kidney injury" OR AKI OR "acute renal failure" OR ARF) AND (sepsis OR septic OR "septic shock") AND (timing OR early OR late OR initiation OR delay) | English language | N= 261 |

**Supplementary Table S2.** The NOS assessment scale for the included cohort studies.

| **ID** | **Cohort studies** | | | | | | | | |
| --- | --- | --- | --- | --- | --- | --- | --- | --- | --- |
|  | **Selection** | | | | **Comparability** | **Outcome** | | | **Quality Score** |
|  | **Representativeness of the exposed cohort** | **Selection of the non-exposed cohort** | **Ascertainment of exposure** | **Demonstration that outcome of interest was not present at start of study** | **Comparability of cohorts on the basis of the design or analysis** | **Assessment of outcome** | **Was follow-up long enough for outcomes to occur** | **Adequacy of follow up of cohorts** |  |
| **Baek 2017** | * | * | * | * | ** | * | * | * | 9/9 |
| **Chon 2012** | * | * | * | * | * | * | * | * | 8/9 |
| **Oh 2012** | * | * | * | * | * | * | * |  | 7/9 |
| **Oh 2016** | * | * | * | * | * | * | * |  | 7/9 |
| **Shum 2013** | * | * | * | * | * | * | * |  | 7/9 |
| **Tian 2014** |  | * | * | * | * | * | * | * | 7/9 |
| **Xiao 2021** |  | * | * | * | * | * | * | * | 7/9 |
| **Yoon 2019** |  | * | * | * | * | * | * | * | 7/9 |
| **Julien 2025** | * | * | * | * | ** | * | * | * | 9/9 |
| **Lee 2024** | * | * | * | * | ** | * | * |  | 8/9 |
| **Li 2026** | * | * | * | * | ** | * | * |  | 8/9 |

**Supplementary Figure S1.** The ROB-2 tool assessment for the included RCTs.

**Supplementary Figure S2.** Funnel plot analysis of 28-days mortality.

**Supplementary Figure S3.** Trim-and-fill analysis plot of 28-days mortality.

**Supplementary Figure S4.** Leave-one-out sensitivity analysis of 28-days mortality.

**Supplementary Figure S5.** Leave-one-out sensitivity analysis of multi-organ dysfunction socre (MODS).

**Supplementary Figure S6.** Leave-one-out sensitivity analysis of intensive care unit (ICU) stay.
